# Social participation in the promoting activity, independence and stability in early dementia (PrAISED), a home-based therapy intervention for people living with dementia: a realist evaluation

**DOI:** 10.1186/s12877-024-05086-y

**Published:** 2024-07-18

**Authors:** Claudio Di Lorito, Kristian Pollock, Vicky Booth, Louise Howe, Sarah Goldberg, Maureen Godfrey, Marianne Dunlop, Rowan H. Harwood, Veronika van der Wardt

**Affiliations:** 1 Department of Primary Care & Population Health, University College London, 1-19 Torrington Place, London, WC1E 7HB UK; 2 School of Health Sciences, University of Nottingham, Nottingham, UK; 3 School of Medicine, University of Nottingham, Nottingham, UK; 4 Nottingham University Hospitals NHS Trust, Nottingham, UK; 5 School of Health and Social Care, University of Lincoln, Lincoln, UK; 6 Philipps University of Marburg, Marburg, Germany

**Keywords:** Social participation, Dementia, Realist evaluation, Physical activity

## Abstract

**Background:**

Interventions promoting social activity may reduce behavioural psychological symptoms and improve quality of life in people living with dementia. This study aimed to identify social benefits for participants living with dementia in the context of Promoting Activity, Independence and Stability in Early Dementia (PrAISED), an exercise intervention programme promoting physical activity and independence in participants living with dementia in England.

**Methods:**

This was a multi-method realist evaluation undertaking secondary analysis of data collected during the PrAISED process evaluation, including qualitative interviews with participants with dementia, caregivers and therapists, personal notes of researchers, and video recordings of therapy sessions. The study consisted of four phases: (1) Setting operational definition of social outcomes in PrAISED; (2) Developing Context, Mechanisms, Outcome (CMO) configurations; (3) Testing and refining CMOs; and (4) Synthesising definitive CMOs into a middle range theory.

**Results:**

Two CMOs were identified. (1) When therapists were able to make therapy sessions engaging and had the caregivers’ support, the participants experienced therapy sessions as an opportunity to achieve goals in areas they were interested in. They also found the sessions enjoyable. This all led to the participants being highly engaged in their social interactions with the therapists. (2) When the participants realised that they were gaining benefits and progress through the PrAISED intervention, such as increased balance, this boosted their confidence in physical ability. It might also reduce caregivers’ risk-aversion/gatekeeping attitude, which in turn would lead to participants’ increased participation in social activities.

**Conclusion:**

The PrAISED intervention supported social participation in participants living with dementia. Under certain circumstances, home-based therapy interventions can be beneficial for social health (regardless of physical health gains). Given the limitations of currently available outcome measures to assess social participation, qualitative methods should be used to explore social health outcomes.

**Supplementary Information:**

The online version contains supplementary material available at 10.1186/s12877-024-05086-y.

## Introduction

Dementia is a neurodegenerative condition characterised by progressive cognitive impairment and loss of functional ability and independence which reduce opportunities to maintain well-being and social activities [1, 2]. Research suggests that exercise and physical activity may have positive effects on functional independence, cognition and psychological health in people living with dementia [3, 4].

Social health, a term which was first developed by Huber et al. [5], and adapted for people living with dementia by the early detection and timely INTERvention in DEMentia (INTERDEM) Social Health Taskforce [6] encompasses: (1) the capacity to fulfil one’s potential and obligations; (2) the ability to manage life with some degree of independence; and (3) participation in social activities. Interventions promoting social activity in people living with dementuia have been developed and tested and have shown some reductions in behavioural and psychological symptoms as well as some improvements in quality of life [7, 8].

The Promoting Activity, Independence and Stability in Early Dementia (PrAISED) intervention was an individually tailored exercise programme to promote physical activity and independence in participants living with mild cognitive impairment (MCI) and early dementia [9] in England. A process evaluation was completed alongside the PrAISED Randomised Controlled Trial (RCT) [10] to identify facilitators and barriers to the intervention and explore mechanisms of effect. The process evaluation found that increased social integration and community activities were a potential but unintended benefit of the PrAISED intervention that warranted further exploration.

Realist approaches are ideal to investigate complex interventions and “social change” [11]. Based on a realist perspective, any programme or intervention is trying to manufacture social change. In fact, “*realist approaches view human agency and social interactions as the very core of the change*” ([12], p125). PrAISED was therefore as much a psychosocial intervention as a physical health intervention, inevitably generating social participation through the interaction of the participant living with dementia, the caregiver(s), and the therapist(s).

### Aim and objectives

This realist evaluation aimed to identify and explain potential social benefits for people living with dementia resulting from interactions with therapists in the context of PrAISED intervention delivery. The objectives were to:

1. Operationally define “social outcome(s)” in the context of PrAISED.

2. Develop Context, Mechanisms, Outcome (CMO) configurations explaining how, under which circumstances and why the social outcomes occurred.

3. Test/refine CMOs.

4. Synthesise definitive CMOs to develop a middle range theory that can be repurposed and reused in different contexts by different health professionals and service providers supporting people living with dementia.

## Methods

### Rationale

This study adopted a multi-method realist approach as described by Pawson and Tilley [13], and is based on the RAMESES II reporting standards for realist evaluations [14]. Realist evaluations provide a systematic yet flexible approach to identify the interacting CMOs generating outcomes within complex interventions, such as PrAISED. As such, they attempt to explain that which is not explicit within a complex intervention, explore generative causality, and develop clear hypotheses as to why a programme is or is not ‘working’ [14]. Realist approaches can also provide a framework for the analysis of multi-method data [15].

### Environment of evaluation

This realist evaluation was based on secondary analyses of data from the PrAISED process evaluation. It was completed by a multidisciplinary research team working on PrAISED including two psychologists, a physiotherapist (PT), an occupational therapist (OT), a sociologist, two geriatricians and two Patient and Public Involvement Engagement (PPIE) members. Regular meetings to develop and undertake the evaluation were held online over 17 months during the PrAISED RCT (September 2021 to January 2023).

### Description of the PrAISED intervention

The intervention is described in detail elsewhere [16]. In brief, PrAISED was a complex intervention delivered in participants’ homes by PTs, OTs and Rehabilitation Support Workers (RSWs). Over 12 months, participants received up to 50 individualised therapy sessions (median 31, IQR 22–40) including supervised exercises, functional activities, support for inclusion in community activities, risk enablement and environmental assessments aiming to improve activities of daily living. Therapist visits were intended to support the exercises and activities, monitor progress and adjust the programme. With the intention of habit formation, therapy visits were tapered, with two visits per week at the start of the intervention programme, slowly being reduced to monthly visits towards the end of the intervention. The typical visit would begin with routine questions around participant’s health and wellbeing, followed by a review of the programme and goals. This was usually a discussion between the therapist, participant living with dementia and their caregiver (if any). The discussion was typically followed by an active part of the session, where the therapist and participant would perform some of the exercises and activities in the programme, and where the therapist introduced new exercises and activities, if appropriate. The caregiver would typically observe. The session would end with a discussion in which the therapist, participant living with dementia and caregiver agreed on some action points for the following session. Due to the Covid-19 pandemic, session delivery was interrupted and partly completed by telephone and videoconferencing (68% was delivered face-to-face, 26% by telephone, 6% by videoconference). In total, participants reported completing on average 482 min of PrAISED-related physical exercise per month (range 0–5310 min; 121 min/week). 

### Recruitment and sampling strategy

The sample of this study included all participants to the PrAISED process evaluation. Recruitment of participants for the process evaluation is described in detail elsewhere [9, 10, 17]. In brief, participants were selected purposively from the PrAISED RCT to represent a diverse range of characteristics (e.g., age, gender, ethnicity, location, relationship and living status). Caregivers of participants living with dementia were also involved in the study, if both they and the participant agreed. Therapists were selected based on different professional roles and study sites.

### Data collection

The following data were used for this realist evaluation:

- Qualitative interviews with participants with dementia (individually or jointly with caregivers, if preferred) and therapists. Interviews were carried out at two time points: start of the intervention and before the end at month 12.
- Personal notes and observations of researchers, routinely compiled on completion of each interview.
- Video recordings of therapy sessions.

### Data analysis

**Fig. 1:**
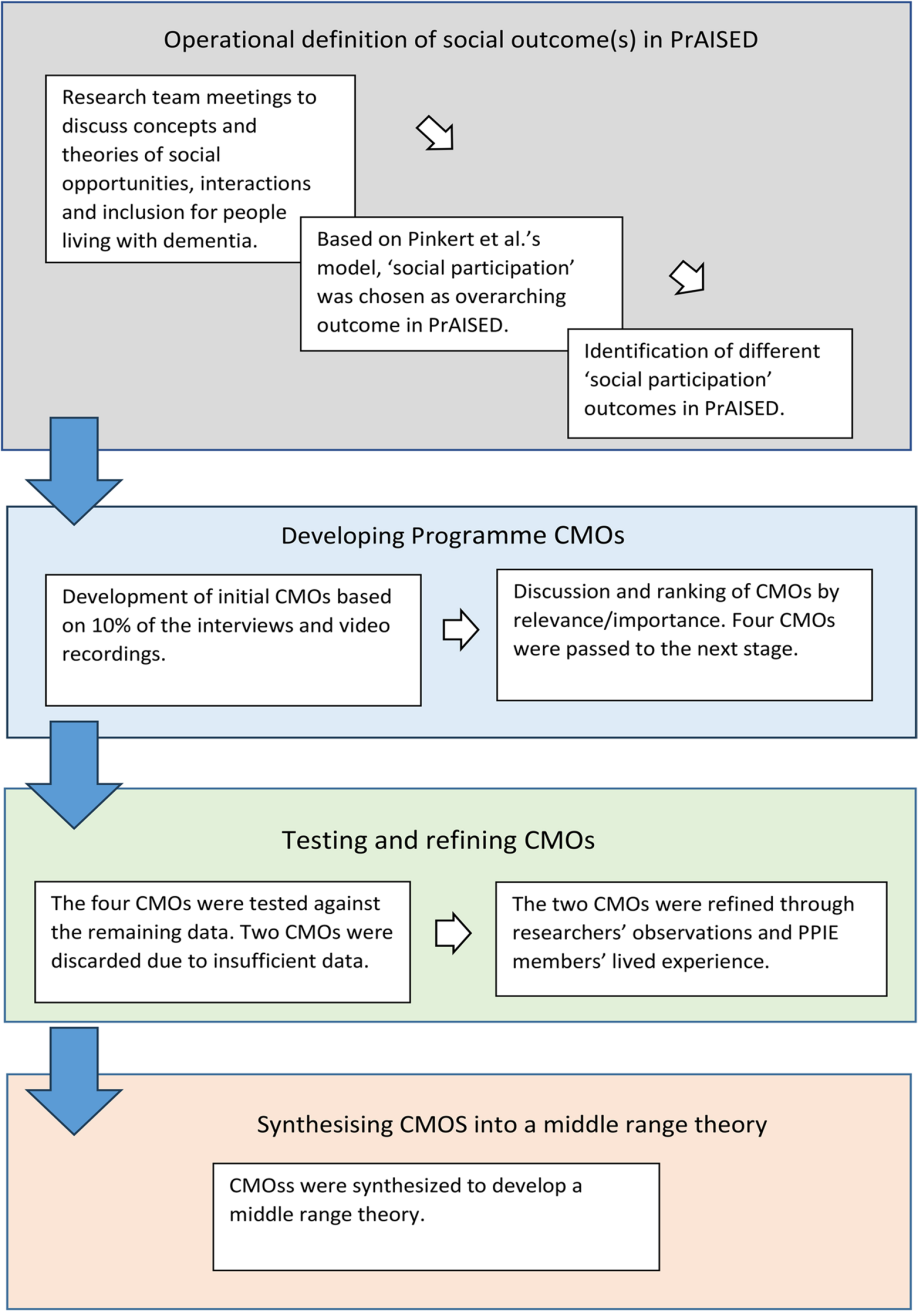
Process of realist evaluation analysis

The realist evaluation consisted of four analytic phases based on the four objectives (Fig. 1): (1) Operational definition of social outcomes in PrAISED; (2) Developing CMOs; (3) Testing and refining CMOs; and (4) Synthesising definitive CMOs into a middle range theory [18].

### Objective 1: operational definition of “social outcome(s)” in PrAISED

This stage occurred through bi-monthly meetings of the realist evaluation team, held between September 2021 and June 2022. In the first meetings, the team discussed concepts and theories of social opportunities, interactions and inclusion for people living with dementia [6, 19]. Based on the conceptualisation of social inclusion by Pinkert et al. [19], the team developed consensus on what is intended by ‘social participation’ and at which ecological levels it occurred in PrAISED (Table 1). For each level, the model also identified the people that the participant living with dementia had social interactions with during the time they were receiving PrAISED.

The term ‘social participation’ was chosen instead of Pinkert’s ‘social inclusion’ as the emphasis in the intervention was on individual interactions rather than on wider societal inclusion. At the micro-level (i.e., the immediate relationships of the participant in PrAISED), social interactions occurred with the primary informal caregiver, all other family members and other people providing in-home support (e.g., paid caregivers). At the meso-level (i.e., people external to the home who came into interaction with the micro-system) were interactions with the therapists delivering PrAISED. At the macro-level (i.e., people in the larger community and society) were interactions with neighbours and friends, attendees at community events, other health care professionals and other people in the community.

The model guided the selection of the Contexts (C’s). These included social interactions and activities initiated and supported through the PrAISED intervention that were meaningful to the person with dementia.

**Table 1:** Model adapted from Pinkert et al. for PrAISED social participation

Ecological level	Micro (The immediate relationships of participants in PrAISED)	Meso (People external to the home interacting with the Micro system in the context of PrAISED)	Macro (Interactions and relations of participant in PrAISED in the community and society)
Examples of social inclusion at ecological level	Interactions with: • Caregiver • Family members • Other in-home supporters	Interactions with: • PTs • OTs • RSWs • Other PrAISED team members	Interactions with: • Neighbours • Friends • Attendees at community events, groups, classes, activities • Health care professionals (e.g., GP) • Other people in the community (e.g., shops, cafes, holidays)

### Objective 2: development of CMOs

The development of the initial CMOs involved iterative secondary analysis of about 10% of PrAISED process evaluation interviews and video recordings of therapy sessions delivered to participants in the intervention arm of the PrAISED RCT. Three members of the realist evaluation team (CDL, VvdW and KP) analysed two interview transcripts and two therapy session videos independently of each other and noted down potential contexts and mechanisms generating participant-level social participation. Social participation as an outcome was the focus of this initial review. Each team member generated ‘rough’ CMOs.

Subsequently, the team discussed these and created seven defined CMOs based on commonalities of rough CMOs through thematic analysis [20]. Two team members with a research background (CDL and VvdW) and two PPIE members with lived experience of caring for someone living with dementia (MG and MD) were then invited to rank the resulting CMOs by relevance/importance. The researchers evaluated the relevance/importance of CMOs based on their reflection of the data and observations throughout the data collection, while the PPIE members based their evaluations on their experience of interviewing participants, and their own lived experience. The input from the two PPIE members ensured that the ranking reflected the views of those affected by dementia. The four CMOs with the highest ranking were passed to the next stage (see appendix 1).

### Objective 3: testing and refining CMOs

The four CMOs that passed the previous stage were tested using the remaining video recordings and interviews. Two coders (CDL and VvdW) analysed the transcripts (each transcript was only looked at by one rater) independently of each other and extracted quotations that corresponded to the C’s, M’s and O’s. Quotations were extracted if they included at least two elements of the CMOs (e.g., mechanism and outcome) and were related to social outcomes. A matrix was created in Microsoft Excel, listing in the rows the Cs, Ms and Os, and in the columns the participants’ anonymised study IDs. The matrix was populated with the extracted quotations. The two PPIE contributors double checked the matrix.

One of five actions was used to reach consensus for each CMOs based on extracted quotations:

1. Confirming the CMOs in their original configuration.
2. Edit the CMOs by eliminating/adding/rephrasing C’s and M’s.
3. Discarding the CMOs altogether when no data was available to test it.
4. Merging the CMOs with another one, when similarities emerged from the data.
5. Separating one CMO into two separate ones, when data showed that they were dissimilar.

Two CMOs remained after the first testing phase, which were passed to a refinement stage. This was carried out through testing the quotations supporting CMOs against personal notes and observations of researchers, routinely compiled on completion of each process evaluation interview. In this iteration, for each of the two CMOs, triangulation between the quotations and the other data sources was undertaken, using the “following a thread” technique [21]. This consisted in using the quotation as starting point and, by “following the thread in the other data sources”, testing the CMOs. In this iteration, the CMOs could not be discarded but the confirmation and refinement process as described above was completed. The resulting CMOs were passed to the PPIE contributors for final review based on their lived experience of dementia.

### Objective 4: synthesising CMOs into a middle range theory

The CMOs were synthesised into a model of interdependent components. Checking the model against research reported in scientific literature, a middle range theory was proposed.

## Results

### Description of sample

CMOs were developed and tested using 24 interviews with participants with dementia and caregivers, 24 with therapists, 14 video recordings of therapy sessions, and personal observations and comments of the interviewers associated with each interview. Participants’ and caregivers’ characteristics, as well as information on their interviews are presented in Appendix 2. Therapists’ characteristics and information on their interviews are reported in Appendix 3. Information on video recordings is presented in Appendix 4.

### Main findings

The initial seven CMOs are reported in Appendix 1. Of the four CMOs ranked the highest and tested using the data and procedures outlined above, two were discarded. In one case quotations evidenced that irregularity/tapering led to less exercising but not that this led in turn to fewer social activities; in the other case, the data did not clarify sufficiently enough the differences in rapport between RSWs and PTs/OTs. The remaining two CMOs were refined and are presented below in their definitive version.

### Positive engagement of the therapist (CMO 1)

*‘When therapists were able to make therapy sessions engaging and had the caregiver’s support (C), the participants experienced therapy sessions as an opportunity to achieve goals in areas they were interested in (M). They also found the sessions enjoyable (M). This all led to the participants being highly engaged in their social interactions with the therapists (O)’*.

This CMO examined how positive engagement with therapists could be achieved in the PrAISED intervention by doing activities that the participants enjoyed. The video recordings of one therapy session showed, for example, the participant and therapist putting up a dartboard with the help of the caregiver and starting a game of darts, for which the therapist had brought the equipment. The interactions between participant, caregiver and therapist showed that they enjoyed the game and the rapport building that this activity generated between the therapist and both participant and caregiver.

The interview data reflected this. Participants living with dementia, caregivers and therapists often acknowledged that the pleasant and motivating sessions were an instrumental mechanism in promoting the participants’ successes and enjoyment of the sessions, which would result in a positive social engagement with the therapists. A parallel or reinforcing mechanism emerging from the interviews was achieving therapy goals, which contributed to the positive experience of the sessions and promoted trust in the therapists. For example, when participants achieved their walking goal, increasing their mobility, the intervention was perceived as having a positive impact on their lives.

The interviews with participants living with dementia and caregivers as well as the interviewers’ observational notes showed that participants and caregivers valued their relationship with the therapists for different reasons. For some it felt close to friendship; others appreciated the therapists’ visits as a means for general support, an opportunity to be active and/or a source of information. The data from the therapists’ interviews reflected their role in the engagement of the participants. In several instances, participants and caregivers would feel quite close to the therapists and became anxious towards the end of the intervention. In the interviews, some therapists expressed concerns about the participants’ potential dependency on them and feelings of being left alone. Some participants living with dementia and caregivers did indeed express a fear towards the end of the intervention of losing contact with the therapists.

### Self-efficacy in people living with dementia (programme theory 2)

*‘When the participants realised that they were gaining benefits and progress through the PrAISED intervention, such as increased balance (C), this boosted their confidence in physical ability (M). It might also reduce caregivers’ risk-aversion/gatekeeping attitude, which in turn would lead to participants’ increased participation in social activities (O).)’*.

This CMO investigated perception of physical improvements in the participants living with dementia as well as their caregivers. Awareness of improvements supported a sense of higher self-efficacy in the person living with dementia. It could also increase caregivers’ confidence that the person they care for would be able to manage the physical challenges associated with social activities, and therefore reduce their gatekeeping attitude and risk aversion. For example, some caregivers expressed in the interviews that in the beginning of the intervention they had been worried about the person they cared for being more physically active. However, seeing the progression in mobility, they had gained confidence that the person could be more active in and out of the house with no risk to safety.

Discussions about positive risk-taking were also part of the intervention and would therefore be initiated by the therapist. The interviews indicated that when both participant living with dementia and caregiver became aware of the improvements in mobility and physical abilities, this was an opportunity for the participant living with dementia to become more active. While it was often clear in the interviews that improvements in physical ability increased confidence, sometimes it might have just been the reassurance of the therapist’s help if things went wrong. These participants might not have had the confidence to go out without the therapist going with them.

Increased confidence could lead the person to undertake more physical activity outside on their own or with support from the therapist and/or garegiver. For some participants, this meant that they could start visiting cafés, shops, or engaging in other social activities such as going to dementia groups. For those who had already routinely engaged in some of these activities before the intervention, it meant that they could continue or expand these activities.

Therapists’ experiences and reflections recorded in the interviews supported this CMO. They regarded the confidence in the participant’s ability to go out and the resulting decrease in risk-aversion or gatekeeping in the caregiver as an important element for joining (social) activities outside the house.

### Interdependence between CMOs: development of a middle range theory

**Fig. 2:**
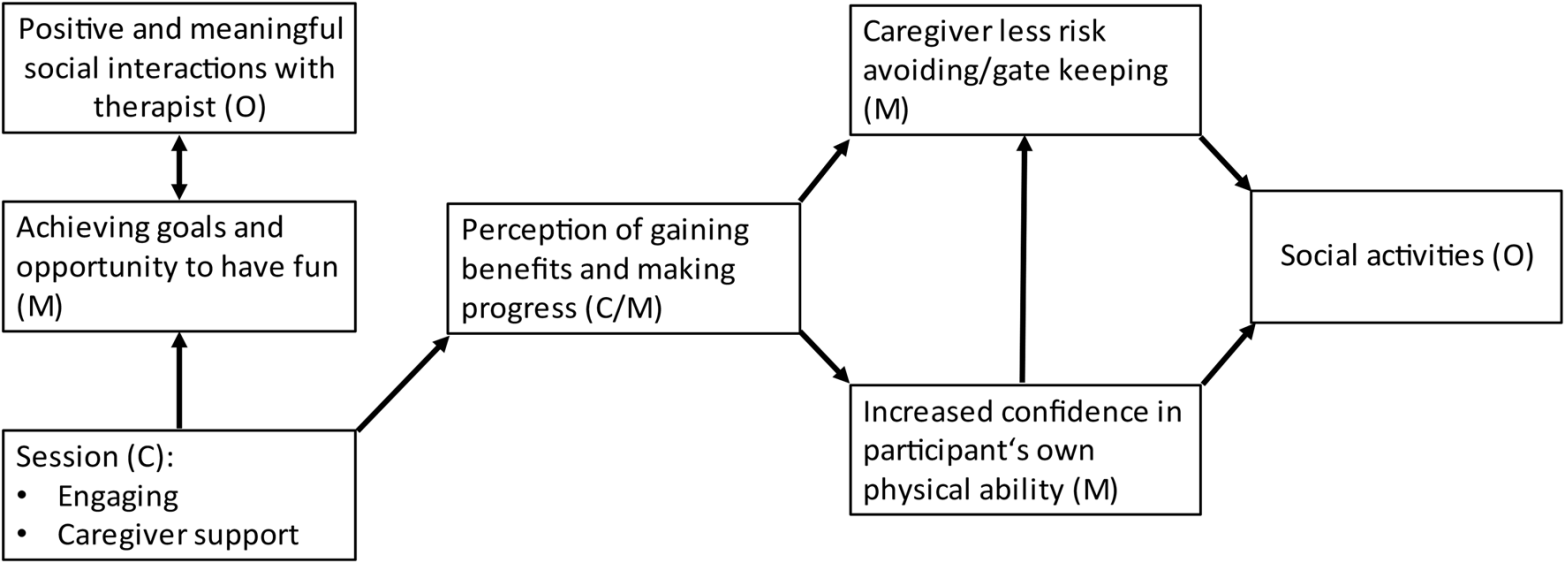
Middle-range theory: Interdependence model of CMOs

While the data supported the two separate components of the CMOs, they also indicated a complex, interdependent relationship between the CMOs. This was synthesized in a middle range theory reported in Fig. 2. In brief, engaging therapy sessions and support by the caregiver were the context for achieving goals and enjoyment, but also for gaining improvements in physical ability, resulting in a perception of benefits. While achieving goals and the opportunity to have fun during the therapy sessions were the mechanisms for positive and meaningful social interactions with the therapist, the perception of gaining benefits and making progress could be considered both a context or a mechanism for the participant to feel more confident in their own ability and for the caregiver to reduce their gate keeping attitudes and encourage social activities.

## Discussion

### Summary of findings

This was a realist evaluation exploring how, under what circumstances and why, the PrAISED intervention could contribute to social participation in participants living with dementia. The concept of social participation for this study was based on a model adapted from Pinkert et al. [19] to reflect different social interactions the participants experienced during their involvement with PrAISED. The adapted model proposed three ecological levels of social participation through interactions within the home (e.g., caregivers; micro level), interactions with the PrAISED team (meso level) and interactions with the wider community (macro level).

The three different levels of this model (micro, meso and macro) are reflected in the results: the outcomes ‘social interaction with therapist’ and ‘social activities in the community’ corresponded with the meso and macro levels of the social participation model. Interactions between the participant living with dementia and their caregiver were often a part of the intervention, but in our study, these were not considered an outcome but rather context and mechanism factors.

The study used secondary data from the PrAISED process evaluation, identified and validated two CMOs, and synthesised these into a middle range theory. The study found that PrAISED contributed to social participation either through engagement in social interactions with the therapists and/or by supporting social activities outside the home. Important contextual factors included the quality and enjoyment of intervention sessions, increased confidence in participant’s own physical abilities, perception of benefits and progress and caregiver support.

The findings showed that therapy sessions, which were supported by the caregiver, and which were perceived as beneficial by the participant led to strong rapport building with the therapist as well as social activities outside the participant’s home. Key mechanisms were achieving goals, enjoying working with the therapist, increasing confidence in one’s own physical abilities and the caregiver accepting the risk of the person with dementia being more active. For some participants and their caregivers, the relationship with the therapist was perceived as vital support, which made them fearful of the end of the intervention (and discontinuation of that support). Some therapists also recognised a risk for dependency on their support.

These findings bring potential evidence to the negative results of the PrAISED RCT [9]. Specifically, they contradict the expectation that therapists could taper support and visits over time and that participants would maintain exercise levels (and associated benefits) because of habit formation. Instead, it appears that continuing engagement of the therapist seemed necessary for participant engagement.

### Comparison with existing literature

Findings from this study reflect the evidence reported in previous literature on the effectiveness of in-person therapy sessions. In our previous study [22] comparing remote versus in-person delivery of PrAISED, we found that while video consultations are acceptable in certain circumstances (e.g., when social distancing is required, in large geographical areas), an in-person approach to rehabilitation better responds to patients’ social needs. In an interview study with older adults using home care services in Norway and Denmark [23] home care visits were also found to bring social life into the house, and could reduce feelings of loneliness. In particular, if the visits were seen as exceeding the expected routine-based care, they had a greater symbolic meaning of inclusiveness for those receiving the care.

This is likely to have played a role in PrAISED intervention visits as well, as therapist visits were on average considerably longer than standard community care visits [71 min vs. 30 min; [9, 24]]. Norvoll et al. [23] indicated that home care visits can initiate social participation opportunities outside the home, which is reflected in our results. These social activities do not necessarily refer to joining local community activities, but can include smaller interactions such as going shopping or visiting a café. Meaningful interactions typically occurred within therapy visits in PrAISED, which was based on the ethos and principles of person-centred care [16]. The opportunity for the participants to be recognised and validated by the therapists in the context of therapy visits enhanced rapport building and social interactions [25].

In relation to the benefits reported from these social interactions, while there is some evidence that social participation and inclusion is associated with health outcomes in older people [26, 27], this could not be confirmed in PrAISED, as the RCT found no difference in health-related outcomes between intervention and control groups [9]. It is also important to acknowledge that not all therapists/participants/caregivers established positive health-enhancing relationships. The videos included some sessions in which the participant and caregiver did not seem at ease, did not enjoy themselves, and at times disengaged. This diversity in experiences calls for caution when suggesting a causal relationship between social interactions/participation and health outcomes in people with dementia.

Different interventions have been designed and tested to increase social participation and inclusion of people living with dementia, often based on the use of technology [7, 8, 28]. However, our evaluation indicated that interventions including regular home visits supporting independence in people living with dementia might lead to social participation as an added benefit, independent of the focus of the intervention. ‘Importance of relationships’ and ‘communication’ have been included in the core outcome items set for non-pharmacological community-based health and social care interventions [29] but these do not encompass all aspects of social participation or inclusion [6, 19]. In light of the evident benefits of social interaction with therapists, this realist evaluation adds to the growing evidence that other less “statistically measurable” outcomes than traditional “health gains” are also important to people living with dementia. Appropriate outcome measures for people with dementia therefore should be developed [30].

The contexts and mechanisms identified within CMOs have been documented before in the literature. The importance of the therapeutic relationship or alliance between a patient and their therapist, for example, was identified in physiotherapy studies [31, 32]. A previous realist review by the team identified the importance of the perception of benefit within exercise to support participation of people living with dementia [33]. Self-efficacy has also been identified as a motivational aid [34]. However, this is the first time these components have been evidenced as supporting social participation in people living with dementia.

### Strengths, limitations and future directions

This study was characterised by certain strengths and limitations. It was rigorously carried out and reported, based on the RAMESES II reporting standards for realist evaluations [14]. It generated novel insights into the social health benefits of intensive home-based exercise interventions for people living with dementia, showing that social participation can be an additional outcome of a physical activity intervention set in in a home environment. Another strength of the study was the consistent PPIE contribution throughout the evaluation. The two PPIE members who were also part of the PrAISED research group and are co-authors on this paper (MD and MG) took part in the meetings to develop the initial CMOs, were co-interviewers in eight interviews with participants and caregivers, and actively contributed to the analysis of the interview and video-recording data. Another strength was ethnographic observation and recording of therapy sessions. Despite the fact that direct observation was time-consuming and lengthy, it allowed us to observe behaviour, attitudes and responses in an ecological and spontaneous way.

The study also had some limitations. The data collection and interview guidelines were not developed for this realist evalutaion, but for the process evaluation of the PrAISED RCT. This might have limited the collection of data relating to social participation, as this was not a focus in the interview topic guides [35]. Social interactions important to the participants might not have been mentioned in the interviews or video recordings. Some of the initial CMOs might have been substantiated if interview questions focussed on social participation.

Ad hoc topic guides might have elicited evidence on less positive social interactions between therapists and participants such as awkward conversations or moments when the participant seemed to feel unsure about an activity. While our data showed examples of these, there was not sufficient evidence to develop specific CMOs regarding negative effects on social participation. Further research to corroborate this evidence would be valuable.

Future research could also focus on people living with different stages of dementia and/or not currently involved in an exercise or physical activity intervention, to explore if intensive face-to-face interventions support social participation independent of a focus on physical activity. Furthermore, it is important to acknowledge that our data represented only a very limited snapshot of the complexity of participants’ and caregivers’ lives. The strict application of contexts, mechanisms and outcomes within the “time-bound” context of PrAISED did not reflect the interactive and changeable nature of social participation.

Future research may also wish to focus on gaining a wider representation of views from people living with dementia from more diverse backgrounds, which were underrepresented in our study. The impact of a multicomponent intervention such as PrAISED may influence people living with dementia in different ways throughout the progressive condition based on different socio-cultural expectations on social relations/interactions. Finally, we only included participants living with dementia who had an informal caregiver. Involving people with no support would be crucial in future research, as they may experience a higher risk of social isolation. Of resounding importance is the continued co-production and involvement of PPIE representatives of people living with dementia from diverse communities within any future study.

## Conclusion

The results of this realist evaluation showed that the PrAISED intervention supported social participation in people living with dementia, indicating that under certain circumstances intensive home-based therapy intervention can be beneficial for social health (regardless of physical health gains). The therapeutic relationship, perception of the benefits and progress of the person living with dementia, support from caregivers, and how these and other contextual components interact, are important factors that can lead to increased social activities in this population. Given the limitations of currently available outcome measures to assess social participation, qualitative methods, including ethnography and conversation analysis, should be used to explore this as part of other social health outcomes. Nonetheless, it is equally important to develop appropriate quantitative assessments for people living with dementia as social participation might also mediate the effect of other parameters. Even if evidence for the effect of physical health interventions in people living with dementia is limited, future research should address social health in intensive home-based therapy interventions.

### Electronic supplementary material

Below is the link to the electronic supplementary material.

Supplementary Material 1

Supplementary Material 2

Supplementary Material 3

Supplementary Material 4

## Data Availability

The datasets generated and analysed during the current study are not publicly available to safeguard anonymity of participants but are available from the corresponding author on reasonable request.
