# Supplementary material for: Social participation in the promoting activity, independence and stability in early dementia (PrAISED), a home-based therapy intervention for people living with dementia: a realist evaluation
Source: BMC Geriatr. 2024 Jul 18;24:615. doi: 10.1186/s12877-024-05086-y (PMC11264791; doi:10.1186/s12877-024-05086-y)
Supplement: Supplementary file 1 — Supplementary Material 1 [file 12877_2024_5086_MOESM1_ESM.docx]

Appendix 1. Initial CMOs developed by the research team and ranking.

| CMO | Ranking by CDL | Ranking by VvdW | Ranking by MG | Ranking by MD | Final ranking* |
| --- | --- | --- | --- | --- | --- |
| When the therapist was able to make therapy sessions engaging and tailored to participants’ preferences and passions (C), the participants experienced therapy sessions not only an opportunity to achieving goals in areas they were interested, but also as an opportunity to have fun (M), which led to them being highly engaged in their social interactions with the therapist (O) | 1 | 3 | 4 | 1 | 2 |
| When therapy visits were not regular or were fragmented because of injuries/incidents (C), the participants experienced reduced motivation to keep engaged in the intervention and increased apathy as well as deconditioning (M), which had an effect on their willingness and ability to find opportunities for social interactions in the community (O) | 4 | 2 | 5 | 5 | 4 |
| When the participants realised that they were gaining benefits and progress through engaging in PrAISED, such as increased balance (C), this boosted their confidence in physical activity (M). It might also reduce caregiver risk-aversion/gatekeeping attitude (M), which in turn would lead to participant’s increased participation in community activities (O) | 2 | 1 | 1 | 3 | 1 |
| When the participants lived alone (C) and/or could not count on the emotional and practical support of the caregiver (C), they struggled to keep motivated to do the exercises in between therapy sessions and achieved fewer benefits from the intervention (M). For example, this might result in them not maintaining their independence, potential missing on social occasions which required mobility/travelling such as community groups and/or holidays/family plans (O) | 6 | 4 | 3 | 4 | 5 |
| When the therapists were able to understand relation dynamics between caregiver and participant (C) and navigate these dynamics to maximise uptake of PrAISED (C), this could not only boost the engagement of the participant in PrAISED (M), but it could also facilitate discussion of taboo subjects and resolution of unspoken conflicts (M). This could act as a vessel for better and more open communication and improved rapport between the participant and the caregiver (O). | 6 | 5 | 2 | 6 | 6 |
| In light of the fact that they spent more time with the participants, RSWs could develop good rapport and trust with participants and caregivers (C). Often, they were perceived as enabler rather than prescribers (C). The sessions would be relaxed and informal (M), and this would make participants less exposed to perceived risk of being “assessed” and more comfortable fully engaging in the social opportunity that the therapy session presented (O). | 3 | 4 | 6 | 2 | 3 |
| In the context of the social restrictions brought by the Covid-19 pandemic national lockdown, PrAISED therapy sessions were carried out remotely through the phone/video conferencing (C). The participants transitioned from considering therapy sessions as both a physical and social occasion to emphasising the social aspects of them (M). They reported looking forward to the regular catch ups with therapists as this ensured (basic) social contact with the outside world at a time where it was dramatically reduced (O). | 5 | 6 | 7 | 7 | 7 |

* CMOs with final ranking 1 to 4 were progressed to the next stage: ‘Testing and refining CMOs’.
