# Supplementary material for: Social participation in the promoting activity, independence and stability in early dementia (PrAISED), a home-based therapy intervention for people living with dementia: a realist evaluation
Source: BMC Geriatr. 2024 Jul 18;24:615. doi: 10.1186/s12877-024-05086-y (PMC11264791; doi:10.1186/s12877-024-05086-y)
Supplement: Supplementary file 2 — Supplementary Material 2 [file 12877_2024_5086_MOESM2_ESM.docx]

Appendix 2. Participants’ and caregivers’ characteristics and interview modality

| Participant ID | Sex | Age | Ethnicity | Living alone? | Caregiver ID | Age | Relationship to participant | Interview modality |
| --- | --- | --- | --- | --- | --- | --- | --- | --- |
| P1 | M | 87 | White | No | C1 | 84 | Wife | Dyadic face-to-face |
| P2 | M | 83 | White | No | C2 | 65 | Wife | Dyadic face-to-face |
| P3 | F | 77 | Black | No | C3 | 46 | Daughter | Dyadic face-to-face |
| P4 | F | 89 | White | Yes | C4 | 57 | Daughter | Dyadic face-to-face |
| P5 | F | 73 | White | No | C5 | 73 | Husband | Dyadic face-to-face |
| P6 | M | 76 | White | No | C6 | 64 | Wife | Dyadic face-to-face |
| P7 | M | 73 | White | No | C7 | 72 | Wife | Dyadic face-to-face |
| P8 | M | 73 | White | No | C8 | 74 | Wife | Dyadic face-to-face |
| P9 | M | 70 | White | No | C9 | 70 | Wife | Dyadic face-to-face |
| P10 | M | 78 | White | No | C10 | 79 | Wife | Dyadic face-to-face |
| P11 | M | 79 | White | No | C11 | 81 | Wife | Dyadic face-to-face |
| P12 | M | 90 | White | No | C12 | 84 | Wife | Dyadic face-to-face |
