# Supplementary material for: Social participation in the promoting activity, independence and stability in early dementia (PrAISED), a home-based therapy intervention for people living with dementia: a realist evaluation
Source: BMC Geriatr. 2024 Jul 18;24:615. doi: 10.1186/s12877-024-05086-y (PMC11264791; doi:10.1186/s12877-024-05086-y)
Supplement: Supplementary file 3 — Supplementary Material 3 [file 12877_2024_5086_MOESM3_ESM.docx]

Appendix 3. Therapists’ characteristics and interview modality

| Therapist ID | Profession | Gender | Type of interview | | Therapist video-recorded | |
| --- | --- | --- | --- | --- | --- | --- |
| T1 | Occupational Therapist | Female | In-person | Yes | |  |
| T2 | Occupational Therapist | Female | In-person | No | |  |
| T3 | Physiotherapist | Female | In-person | Yes | |  |
| T4 | Rehabilitation Support Worker | Female | In-person | Yes | |  |
| T5 | Occupational Therapist | Female | In-person | Yes | |  |
| T6 | Physiotherapist | Male | In-person | Yes | |  |
| T7 | Rehabilitation Support Worker | Female | In-person | Yes | |  |
| T8 | Rehabilitation Support Worker | Female | In-person | Yes | |  |
| T9 | Rehabilitation Support Worker | Female | In-person | No | |  |
| T11 | Rehabilitation Support Worker | Female | In-person | No | |  |
| T12 | Occupational Therapist | Female | In-person | Yes | |  |
| T13 | Rehabilitation Support Worker | Female | In-person | Yes | |  |
| T14 | Occupational Therapist | Female | In-person | No | |  |
| T16 | Physiotherapist | Female | In-person | Yes | |  |
| T17 | Occupational Therapist | Female | In-person | No | |  |
| T18 | Rehabilitation Support Worker | Male | In-person | Yes | |  |
| T19 | Physiotherapist | Female | In-person | Yes | |  |
| T20 | Physiotherapist | Female | In-person | Yes | |  |
| T21 | Physiotherapist | Female | In-person | Yes | |  |
| T23 | Physiotherapist | Female | In-person | No | |  |
| T24 | Rehabilitation Support Worker | Female | In-person | No | |  |
| T25 | Rehabilitation Support Worker | Male | In-person | No | |  |
| T27 | Occupational Therapist | Female | In-person | No | |  |
| T28 | Rehabilitation Support Worker | Female | In-person | No | |  |
