# Supplementary material for: Social participation in the promoting activity, independence and stability in early dementia (PrAISED), a home-based therapy intervention for people living with dementia: a realist evaluation
Source: BMC Geriatr. 2024 Jul 18;24:615. doi: 10.1186/s12877-024-05086-y (PMC11264791; doi:10.1186/s12877-024-05086-y)
Supplement: Supplementary file 4 — Supplementary Material 4 [file 12877_2024_5086_MOESM4_ESM.docx]

Appendix 4. Therapist/participant dyads in video recordings

| Therapist | Designation | Site | Participant Number |
| --- | --- | --- | --- |
| T1 | OT | Nottingham | P13 |
| T3 | Physio | Nottingham | P14 |
| T4 | RSW | Nottingham | P15 |
| T5 | OT | Derby | P16 |
| T6 | Physio | Derby | P17 |
| T7 | RSW | Derby | P18 |
| T8 | RSW | Derby | P19 |
| T12 | OT | Lincoln | P20 |
| T13 | RSW | Lincoln | P6 |
| T16 | Physio | Lincoln | P20 |
| T18 | RSW | Bath | P21 |
| T19 | Physio | Bath | P12 |
| T20 | Physio | Bath | P11 |
| T21 | Physio | Nottingham | P22 |
